# Effects of *Euphorbia humifusa* Extract on Nutrient Digestibility, Diarrhea, Serum Biomarkers, and Anti-Inflammatory Mechanisms in Preweaned Calves

**DOI:** 10.3390/ani15202979

**Published:** 2025-10-15

**Authors:** Chuntao Zhang, Zhongying Xing, Wenxiao Feng, Yan Tu, Qiyu Diao

**Affiliations:** 1 Key Laboratory of Feed Biotechnology of Ministry of Agriculture and Rural Affairs, Feed Research Institute of Chinese Academy of Agricultural Sciences, Beijing 100081, China; z13598192302@163.com (C.Z.); tuyan@caas.cn (Y.T.); 2 Beijing Shounong Livestock Development Co., Ltd., Beijing 100081, China

**Keywords:** calf, calf growth, milk replacer, plant extract, *Euphorbia humifusa* extract, network pharmacology, anti-inflammatory

## Abstract

**Simple Summary:**

Calves’ healthy growth and strong immunity are important for livestock farming, but finding safe, effective feed additives to support these needs remains a key concern. This study focused on the extract from a plant called *Euphorbia humifusa* extract (EHE) to see if it could work as a new feed additive. First, we identified the main helpful substances in EHE: polyphenols (most of which are flavonoids, a type of compound with health benefits) and sugars. We then tested adding EHE to milk replacers (a common feed for calves) and found it clearly improved calf growth—with better results when using the right amount. We also studied how EHE works: it acts on several key molecules in calves’ bodies and affects processes related to immunity, stress, and cell health. It also reduces inflammation and helps fight viruses by blocking a pathway called NF-κB. This research provides a safe feed additive option to boost calf health, supporting better livestock farming.

**Abstract:**

Early-life rearing of animals is critical for their lifelong productivity, health, and the quality/safety of livestock products. EHE, a feed additive with growth-promoting, antibacterial, and immunity-enhancing properties, was tested for effects on preweaned calves. Forty-eight calves (42.18 ± 0.61 kg) were randomly assigned to four groups (*n* = 12/*group*), fed milk replacer with 0 (CON), 400 (A), 800 (B), or 1200 (C) mg/d EHE for 60 d (after 6 d adaptation). Growth, nutrient digestibility, serum biomarkers, rumen fermentation, and diarrhea incidence were measured; network pharmacology was used to analyze EHE’s targets. Results: Group C had 14.09% higher body weight gain (52 vs. 45 kg, *p* < 0.05), higher dry matter intake/digestibility, and increased acid detergent fiber digestibility vs. CON. Group C had reduced diarrhea frequency, tended to have lower rumen acetate-to-propionate ratio, and had higher early rumen volatile fatty acids (VFA). At d 66, Groups B and C had reduced serum IL-6/IL-8 (*p* < 0.05). Network pharmacology identified 256 anti-inflammatory targets (e.g., BCL2, IL6) involved in apoptosis/inflammatory pathways. Conclusion: 1200 mg/d EHE optimally improves calf growth, digestibility, and anti-inflammatory status.

## 1. Introduction

China boasts the largest population of cattle and sheep worldwide. Ruminant animal husbandry serves as a vital livelihood sector in the country, deeply intertwined with people’s daily lives and socioeconomic development. As the foundational cohort for adult livestock populations, the targeted rearing of young ruminants directly influences the growth trajectories and product quality of mature animals. Optimal management of juvenile cattle and sheep during this developmental phase establishes a robust foundation for subsequent livestock slaughter efficiency and economic returns. Collectively, the standard of early-life rearing practices has emerged as a pivotal determinant shaping the sustainable development of the cattle and sheep husbandry sector [1].

The immune system of neonatal calves remains genetically immature, with incomplete functional development of multiple organ systems and tissues. This results in inherently weak immunocompetence, rendering calves highly susceptible to environmental perturbations. The synergistic interplay of these multi-factorial challenges imposes significant burdens on neonatal calf rearing management [2].

Consequently, science-based rearing practices serve as indispensable strategies to promote the healthy development of juvenile livestock and enhance the genetic quality of China’s livestock populations. Since the 20th century, antibiotics have been ubiquitously employed as feed additives, playing a pivotal role in maintaining the health and promoting the growth of livestock and poultry. However, prolonged reliance on antibiotic use has precipitated critical challenges, including drug residue accumulation and the emergence of antimicrobial resistance, which pose significant risks to public health and have consequently sparked widespread concerns regarding their safety profiles [3,4].

Plant-derived bioactive compounds, emerging as a novel category of green feed additives, have garnered substantial research attention owing to their inherent multi-functionality, low toxicity profiles, and non-antimicrobial resistance characteristics, thereby exhibiting significant potential for intensive development and application in modern animal husbandry systems [5,6].

EHE, derived from the dried aerial parts of *Euphorbia humifusa Willd*. or *Euphorbia maculata* L. (family *Euphorbiaceae*), is a traditional herbal medicine known by various names, including Caoxuecao, Xuejianchoucao, and Tiexiancao. Abundant in bioactive compounds, particularly flavonoids such as quercetin, kaempferol, rutin, luteolin, and apigenin, EHE exhibits a diverse array of pharmacological properties, encompassing antibacterial, anti-inflammatory, antitumor, and immunomodulatory activities [7]. Our research group’s prior investigations demonstrated that dietary supplementation with plant-derived bioactive compounds, including flavonoids (e.g., *baicalin*) and polyphenols (e.g., *rutin*, *paeoniflorin B4*, *resveratrol*), effectively enhanced growth performance in preweaned calves, decreased the incidence and severity of preweaning diarrhea, and improved lactation performance in heat-stressed dairy cows [6] and preweaned cows [8], Similar benefits were observed in broiler chickens, where such supplements increased body weight gain (BWG), average daily feed intake, and feed conversion efficiency while reducing disease prevalence [9]. Notably, Zhang Lei et al. [10] reported that EHE intervention significantly mitigated disease activity index (DAI) scores, weight loss, colonic ulceration, intestinal epithelial barrier disruption, and inflammatory cytokine expression in mice with ulcerative colitis.

Based on the biological properties of *Euphorbia humifusa*, we hypothesize that its extract (EHE) can enhance growth performance, modulate immune responses, and promote physiological health in calves. Thus, this study was designed to use preweaning calves as research subjects to accomplish the following: ① evaluate the effects of EHE on growth performance, nutrient digestibility and metabolism, and serum biomarkers in preweaning calves; ② employ network pharmacology combined with molecular docking approaches to elucidate the anti-inflammatory mechanism of EHE, thereby providing a novel research framework for systematic analysis of EHE’s multi-component, multi-target, multi-pathway synergistic regulatory pattern.

This investigation aims to explore the application potential of EHE in calf rearing and furnish scientific evidence for its translation into practical animal husbandry practices.

## 2. Materials and Methods

### 2.1. Trial Duration and Location

The trial was conducted at Beijing Sanyuan Green Lotus Dairy Cattle Breeding Center from September to November 2024, lasting 66 days, including a 6-day pre-trial period and a 60-day main trial period.

### 2.2. Trial Materials

*Euphorbia humifusa* extract (EHE), a brown powder, was obtained from its dried whole herbs and is rich in a variety of active ingredients. A targeted metabolomics approach method was used to determine its active ingredients [11]; the data are shown in Table 1. Specific components are listed in Table 1.

Milk replacer (MR) was provided by the Beijing Precision Animal Nutrition Research Center.

### 2.3. Trial Animals and Experimental Design

A total of 48 clinically healthy Holstein female calves (2–3 days old, 42.18 ± 0.61 kg) were selected for the trial. These calves all come from the same pasture and were born within a week. Calves were randomly assigned to four treatments (12 calves/treatment), fed twice daily with MR supplemented with 0 mg (control, CON), 400 mg (Group A), 800 mg (Group B), or 1200 mg (Group C) of EHE. The specific feeding pattern is listed in Table 2. Calves were separated from their dams immediately after birth and received 4 L of colostrum via gavage within 1 h of birth, followed by a second gavage within 24 h. Following standard operating procedures on experimental dairy farms, the Immunoglobulin G (IgG) concentration in colostrum was determined using a handheld Brix refractometer (RHB-32, ATC, Inc., YHEQUIPMENT CO., LIMITED, Address: NO.180 Banxuegang big road, Longgang Area, Shenzhen City, China; readings > 22% Brix) [12]. Subsequently, within 72 h postpartum, the serum total protein concentration in calves was assessed using a separate handheld Brix refractometer (HT312, ATC, Inc., HT tech Limited, HongKong, China; range: 0–12 g/dL). All experimental calves exhibited serum total protein concentrations > 5.5 g/dL, meeting the established adequacy criterion [13]. Calves were moved from the calving pen to experimental hutches at 3 days of age. From days 3 to 6, calves were fed a mixture (143 g/L) of on-farm fresh milk and trial MR twice daily (2 L per feeding), with MR inclusion increasing by 1/9 per feeding until full transition to MR by day 7. Nutritional compositions of MR and starter feed are shown in Table 3.

### 2.4. Sample Collection and Measurement

#### 2.4.1. Growth Performance

The offered and refused amounts of milk replacer (MR) and starter feed were recorded daily to calculate the previous day’s actual feed intake. Dry matter intake (DMI), body weight gain (BWG), and feed conversion rate (F/G) were then calculated based on these records.

Samples of MR and starter feed were collected every 2 weeks. For fecal samples, after collection, they were immediately stored at −20 °C to preserve nutrient composition until they were transported to the laboratory for analysis. The dry matter content was determined according to the methods in Feed Analysis and Feed Quality Testing Technology, including crude protein (method 990.03), crude fat (method 2003.05), crude ash (method 942.05), calcium (method 985.01), and phosphorus (method 985.01). Neutral detergent fiber (NDF) and acidic detergent fiber (ADF) were measured using Van Soest’s method after treatment with alpha amylase [14].

#### 2.4.2. Nutrient Digestibility

After the formal experiment was completed (66–73 d), apparent nutrient digestibility coefficients were determined via total fecal and urine collection over 5 consecutive days. Calves (*n =* 5 *per treatment group*, selected for similar body weights post-feeding trial) were housed in individual metabolism crates to enable separate excretion collection. Following a 3-day adaptation period, a 5-day formal collection period was conducted.

Fecal collection protocol: Total feces were weighed daily, homogenized, and sampled (10% aliquot). Samples were treated with 10 mL of 10% H_2_SO_4_ per 100 g fresh feces to minimize nitrogen volatilization. Treated samples were stored at −20 °C until analysis. Prior to analysis, samples were dried at 65 °C for 48 h, equilibrated to room temperature (24 h), and ground to pass a 1 mm sieve.

Urine collection protocol: Daily urine output was collected in containers pre-charged with 200 mL of 10% H_2_SO_4_. The total volume of urine in each container was measured using a 5 L graduated cylinder (accuracy: ±10 mL), then thoroughly mixed, and subsampled (100 mL aliquot). Aliquots were stored at 4 °C during collection and transferred to −20 °C for preservation.

#### 2.4.3. Rumen Fluid Sampling

Following the formal experimental period, rumen fluid was collected from the oral cavity via rumen tubes (length: 100 cm, inner diameter: 8 mm; model: RT-08, Beijing Animal Husbandry Equipment Co., Ltd. Beijing, China) 2 h prior to morning feedings. A manual vacuum pump (model: VP-01, Shanghai Biological Instrument Co., Ltd. Shanghai, China) was used to aspirate approximately 50 mL of rumen fluid per calf, which was immediately filtered through four layers of gauze, and pH was measured using a portable meter (Testo 206 pH2, Testo SE & Co. KGaA, Titisee-Neustadt, Germany). Subsequently, the remaining samples were aliquoted into 15 mL centrifuge tubes and stored at −20 °C for subsequent analysis of rumen fermentation parameters.

#### 2.4.4. Collection and Scoring Records of Fecal Samples

Fecal sample collection: Fresh feces were collected from 24 calves (6 per group) at 30 and 60 days of age. Feces were collected using the rectal stimulation method, which involves using sterile gloves to directly palpate and collect feces from the rectum [15]. Fresh fecal samples were collected in sterile 2 mL cryovials (Corning, Corning Inc., Reynosa, Mexico) and immediately frozen in liquid nitrogen for subsequent fermentation parameter analysis.

Fecal score: Fecal scoring was conducted daily, and the fecal score was evaluated on a 4-point scale based on the fluidity and viscosity of feces [16] (Table 4), where fecal score 0 = normal viscosity, 1 = semi-formed or pasty, 2 = loose feces, and 3 = watery feces. Every morning from 9 to 11 o’clock after feeding, the fresh feces in the calf island were observed for evaluation. If the fecal score was ≥2 for two consecutive days, it could be determined as diarrhea.

#### 2.4.5. Serum Sample Collection and Determination

At the age of 36 and 66 days, 10 mL of blood was collected through the jugular vein 2 h before morning feeding. The serum was immediately centrifuged at 2000× *g* 4 °C for 20 min, and the supernatant was divided into 1.5 mL centrifuge tubes and stored at −20 °C for future use to determine the serum’s anti-inflammatory ability and immune function. The bovine ELISA kit (Beijing Jinhai Keyu Biotechnology Development Co., Ltd. Beijing, China) was used to measure tumor necrosis factor-α (TNF-α), interleukin-6 (IL-6), interleukin-8 (IL-8), and interleukin-10 (IL-10). The intra-batch Coefficient of Variation (CV) of the ELISA kit for TNF-α, IL-6, IL-8, and IL-10 was less than 10%, and the inter batch CV was less than 15%. The pro-inflammatory cytokines TNF-α, IL-6, and IL-8, and the anti-inflammatory cytokine IL-10 were selected as biomarkers to assess the systemic inflammatory and immune status of the calves. Measuring these cytokines helps evaluate the potential of EHE to modulate the immune response, which is crucial for calf health, especially during the preweaning period when they are susceptible to infections and inflammation that can impact growth performance.

### 2.5. Network Pharmacology Analysis

#### 2.5.1. Target Collection

Using the Traditional Chinese Medicine Systems Pharmacology Database (TCMSP), with oral bioavailability (OB) ≥ 30% and drug likeness (DL) ≥ 0.18 as screening criteria, effective compounds and corresponding targets of EHE were retrieved and obtained. After screening, they were stored in the Uiprot protein database (https://www.Uniprot.Org, accessed on 16 December 2024) which converts the protein targets targeted by compounds into standardized gene identifiers. Using “inflammation” as the search term, we searched for antioxidant related targets in the OMIM (https://www.omim.org/, accessed on 18 December 2024), GeneCards (https://www.genecards.org/, accessed on 18 December 2024), PharmGKB (https://www.drugsnav.com/tag/so-PharmGKBshujuku, accessed on 19 December 2024), and TTD databases (https://db.idrblab.net/ttd/, accessed on 21 December 2024). After deduplication, we took the intersection and drew a Venn diagram for EHE anti-inflammatory treatment.

#### 2.5.2. Construction of EHE Antioxidant Target Gene Network

Protein–protein interaction (PPI) analysis was performed on the intersection target STRING database of EHE and inflammation, the above information was imported into Cytoscape 3.10.3 for topological analysis, and a visual interaction relationship between the EHE active ingredients and antioxidant targets was constructed, as well as for the EHE anti-inflammatory key targets.

#### 2.5.3. GO and KEGG Pathway Enrichment Analyses

GO functional enrichment analysis and KEGG pathway analysis [17] were mainly conducted using R language (v4.3.0); the WeChat platform (v2024.10, https://www.bioinformatics.com.cn/, Newcore, Shanghai, China) was used for auxiliary verification to ensure result consistency.

### 2.6. Statistical Analysis

The data were analyzed using SAS 9.2 for one-way ANOVA and LSD for multiple comparisons. The results are presented as mean and standard error (SEM), with *p* < 0.05 indicating significant differences and *p* > 0.05 indicating insignificant differences. For variables related to EHE dose (e.g., growth performance, nutrient digestibility), the relationships between EHE dose and each response variable were analyzed using linear, quadratic, and cubic orthogonal contrasts to evaluate dose-dependent patterns. For variables measured at different age stages (6–36 d and 37–66 d, e.g., fecal score, serum cytokines), a repeated-measures ANOVA was used to include time effect, treatment effect, and time × treatment interaction in the statistical model. Multiple comparisons were performed using LSD.

## 3. Results

### 3.1. Effects of EHE on Growth Performance and Nutrient Digestibility of Preweaned Calves

Table 5 shows that EHE supplementation promoted final body weight and total BWG in calves. Linear contrast analysis revealed that total BWG increased linearly with increasing EHE dose (*p* = 0.028). Specifically, Group C (highest EHE dose) had a 16.7% higher total BWG than the CON group (52.50 vs. 45.00 kg, *p* < 0.05), and the ADG of Group C calves was 104 g/d higher than that of CON calves, with this difference being significant during the 37–66-day period (*p* < 0.05). This confirms a dose-dependent growth-promoting effect of EHE, rather than just an effect at the highest dose. In contrast, Group B showed no significant difference in total BWG compared to CON (*p* > 0.05). Additionally, EHE supplementation in MR significantly increased DMI in a dose-dependent manner; DMI was positively correlated with total BWG, with Group C having the highest DMI (1321.40 kg), which was 18.28% significantly higher than CON (1117.16 kg, *p* < 0.05).

Table 5 further demonstrates that EHE exerts an impact on nutrient digestibility. Specifically, the digestibility of dry matter (DM), crude protein (CP), ether extract (EE), neutral detergent fiber (NDF), and acid detergent fiber (ADF) in the diet increased with escalating dietary EHE supplementation levels. Among these parameters, the digestibility of DM and ADF in Group C reached 74.31% and 74.31%, respectively, significantly higher than those of the CON (69.15%, 28.98%). While EHE tended to improve the digestibility of CP, EE, and NDF, no significant differences were detected among groups (*p* > 0.05).

### 3.2. Effect of EHE on Rumen Fermentation Parameters in Preweaned Calves

As Table 6, compared with the CON group, supplementation of EHE in MR did not significantly affect rumen pH or Total Volatile Fatty Acids (TVFA) in calves before weaning (*p* > 0.05). However, it induced a trend toward decreased acetate-to-propionate (A/P) ratio and isobutyric acid concentration (0.05 < *p* < 0.1), with the most notable trend observed in Group C (A/P: 1.22 vs. 1.63 in CON; isobutyric acid: 0.50% vs. 0.45% in CON).

### 3.3. Effect of EHE on Fecal Score and Diarrhea Rate of Preweaned Calves

At 6–36 days and 37–66 days of age, the fecal score and diarrhea rate of calves in Group C were significantly lower than those in CON (*p* < 0.05). Although a numerical decreasing trend in fecal score and diarrhea rate was observed with increasing EHE dose, the linear trend was not significant (*p* > 0.05; Table 7), so no conclusion of a dose-dependent decrease can be drawn based on linear contrast results. Quadratic and cubic contrast analyses were excluded because the study’s dose gradient (400/800/1200 mg/d) and sample size (*n =* 12 *per group*) did not provide sufficient statistical power to detect non-linear relationships, and the primary objective was to evaluate linear dose responses relevant to practical livestock applications.

### 3.4. Effect of EHE on Fecal Fermentation Parameters of Preweaned Calves

As shown in Table 8, there was no significant difference in fecal pH among the groups of calves (*p* > 0.05). From day 6 to day 36 of the experiment, total VFA (concentration, μmol/g feces), acetic acid (molar percentage, %), and isobutyric acid (molar percentage, %) were significantly higher in Group C than in CON (*p* < 0.05). However, with increasing age, the concentration of isobutyric acid in Group C decreased (*p* < 0.05).

### 3.5. The Effect of EHE on Serum Cytokines in Preweaned Calves

As detailed in Table 9, although EHE did not modify serum TNF-α and IL-10 over the total study period (*p* > 0.05), it significantly reduced serum IL-6 (*p* = 0.028) and IL-8 (*p* = 0.075) concentrations at 37–66 d, which may contribute to improved growth performance. Additionally, increased nutrient digestibility (e.g., DM, ADF) further supports the growth-promoting effect of EHE.

### 3.6. Network Pharmacology Analysis

#### 3.6.1. Acquisition of EHE Targets, Antioxidant-Related Targets, and Potential Action Targets

Using the TCMSP database, a total of 38 active components were initially retrieved. Subsequent screening with criteria of oral bioavailability (OB) ≥ 30% and drug-likeness (DL) ≥ 0.18 yielded 13 key active components. Target genes associated with these components were identified via the Uniprot database, followed by standardization to obtain 256 target genes corresponding to 20 active components. After deduplication, eight potential core targets were shortlisted.

**Figure 1:**
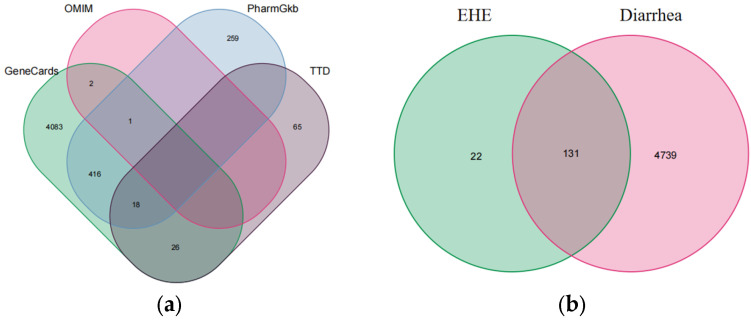
Disease-related targets and drug–disease target intersection: (**a**) Venn diagram of inflammation-related targets retrieved from the OMIM, PharmGKB, TTD, and GeneCards databases (416 common targets were obtained after deduplication); (**b**) Venn diagram of the intersection between EHE-related targets (131) and diarrhea-related targets (4739), with 22 common targets (these are potential targets of EHE for alleviating calf diarrhea).

In parallel, an extensive literature mining using “inflammation” as the keyword was conducted across the OMIM, GeneCards, PharmGKB, and TTD databases to retrieve inflammation-related target genes, Among them, 259 exist simultaneously in OMIM, PharmGKB, and TTD (1a). Data from each database were processed using the R language Venn package to eliminate duplicates, identify intersecting targets, and construct a drug–disease Venn diagram depicting the overlap between EHE-related targets and inflammation-associated genes (Figure 1).

#### 3.6.2. PPI Network Construction Analysis and Core Target Screening

**Figure 2:**
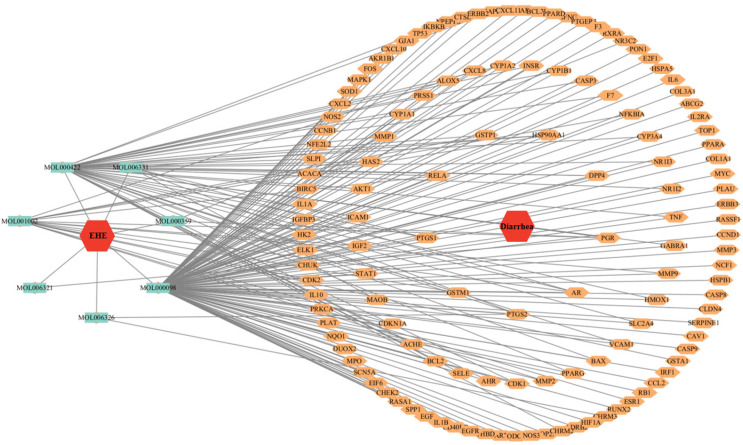
EHE active component–anti-inflammatory target network diagram: Nodes represent EHE active components (red nodes) and anti-inflammatory targets (blue nodes); edges represent the interaction between active components and targets. The size of nodes is positively correlated with the number of interactions (degree value), indicating that components with larger nodes and targets with larger nodes play a core role in the network.

A total of 20 EHE active components and 131 inflammation-related genes were uploaded to the Cytoscape database to construct an active component–target network diagram (Figure 2). The network visualization revealed that EHE exerts anti-inflammatory effects through a multi-component, multi-target regulatory pattern [18].

**Figure 3:**
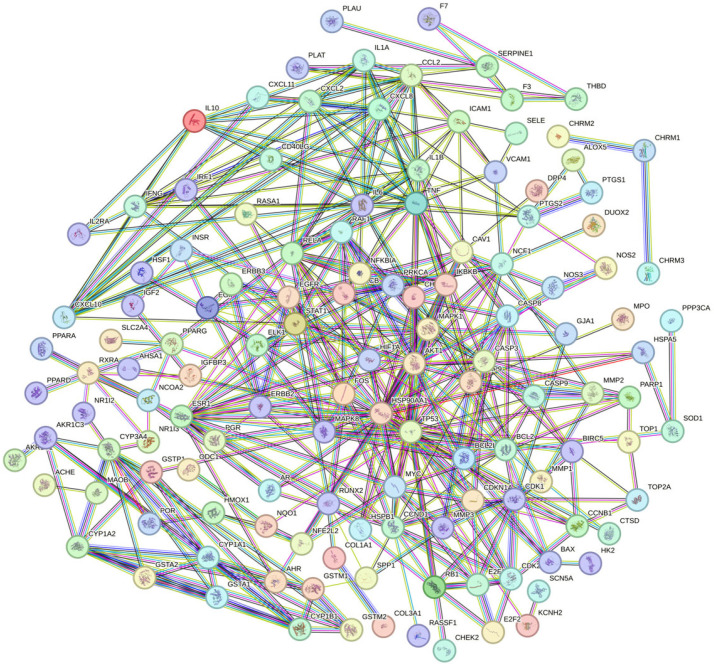
PPI network diagram of EHE anti-inflammatory target proteins: Nodes represent target proteins (132 in total); edges represent protein–protein interactions (1755 in total). The minimum combined score is 0.9 (STRING database), and disconnected nodes are removed. The color depth of nodes is positively correlated with the degree value (interaction frequency), with core targets shown in dark red.

**Figure 4:**
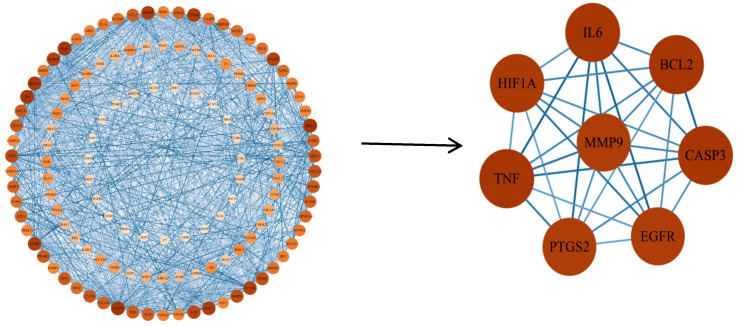
Topological analysis diagram of EHE core anti-inflammatory targets: The diagram shows the top 8 core targets (BCL2, IL6, CASP3, EGFR, MMP9, PTGS2, TNF, HIF1A) screened by the CytoNCA plugin (Cytoscape 3.10.3). The x-axis represents the degree value (number of protein interactions), and the y-axis represents the betweenness centrality (ability to mediate interactions between other nodes). These targets have high degree and betweenness centrality, indicating their key role in the anti-inflammatory network.

Oxidative stress-related targets of EHE’s primary active components were subsequently imported into the STRING database for protein–protein interaction (PPI) analysis. The screened EHE active components and disease-relevant key targets were visualized in Cytoscape 3.10.3 to establish a component–target interaction network. By setting a minimum combined score of 0.9 and removing disconnected nodes, a PPI network comprising 132 nodes and 1755 edges was generated (Figure 3). Using the CytoNCA plugin in Cytoscape to analyze the PPI network (Figure 4), key targets identified for EHE-mediated alleviation of oxidative stress in calves included BCL2, IL6, CASP3, EGFR, MMP9, PTGS2, TNF, and HIF1A.

#### 3.6.3. GO Function and KEGG Pathway Enrichment Analysis

**Figure 5:**
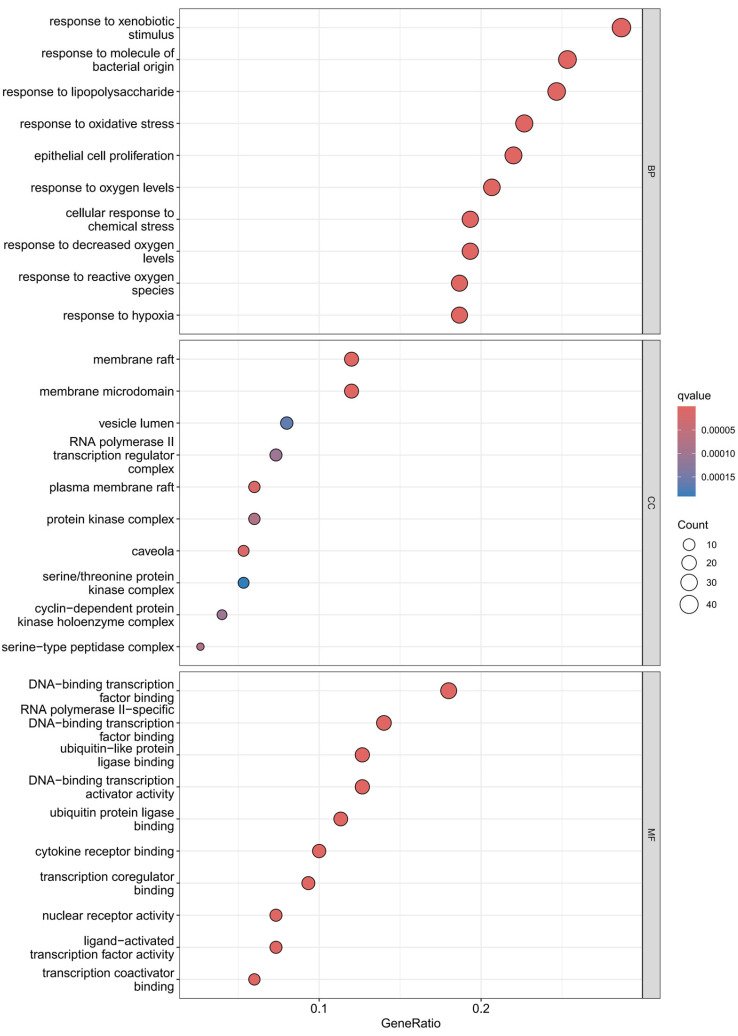
GO functional enrichment analysis diagram: The left panel shows the top 10 enriched biological processes (*p* < 0.05), including response to molecules of bacterial origin and response to oxidative stress; the right panel shows the top 10 enriched cellular components (*p* < 0.05), including membrane raft and collagen-containing extracellular matrix. The x-axis represents GeneRatio (proportion of target genes in the total genes of the pathway), and the y-axis represents the pathway name.

Using the R language, GO enrichment analysis was performed on the target genes, yielding 131 enriched GO entries from 20 targets (Figure 5). With *p* < 0.05 set as the significance threshold, the top 10 most significantly enriched pathways were selected for visual analysis. The GO analysis primarily annotated biological processes related to immune and stress responses, including response to molecules of bacterial origin, response to lipolysis, response to xenobiotic stress, response to oxidative stress, response to oxygen levels, response to reduced oxygen levels, response to hypoxia, response to reactive oxygen species, and cellular response to chemical stress. Additionally, pathways involving epithelial cell proliferation/differentiation and cell structure were identified, such as membrane raft, membrane microdomain, external side of plasma membrane, collagen-containing extracellular matrix, serine/threonine protein kinase complex, cyclin-dependent protein kinase holoenzyme complex, and serine-type peptidase complex.

**Figure 6:**
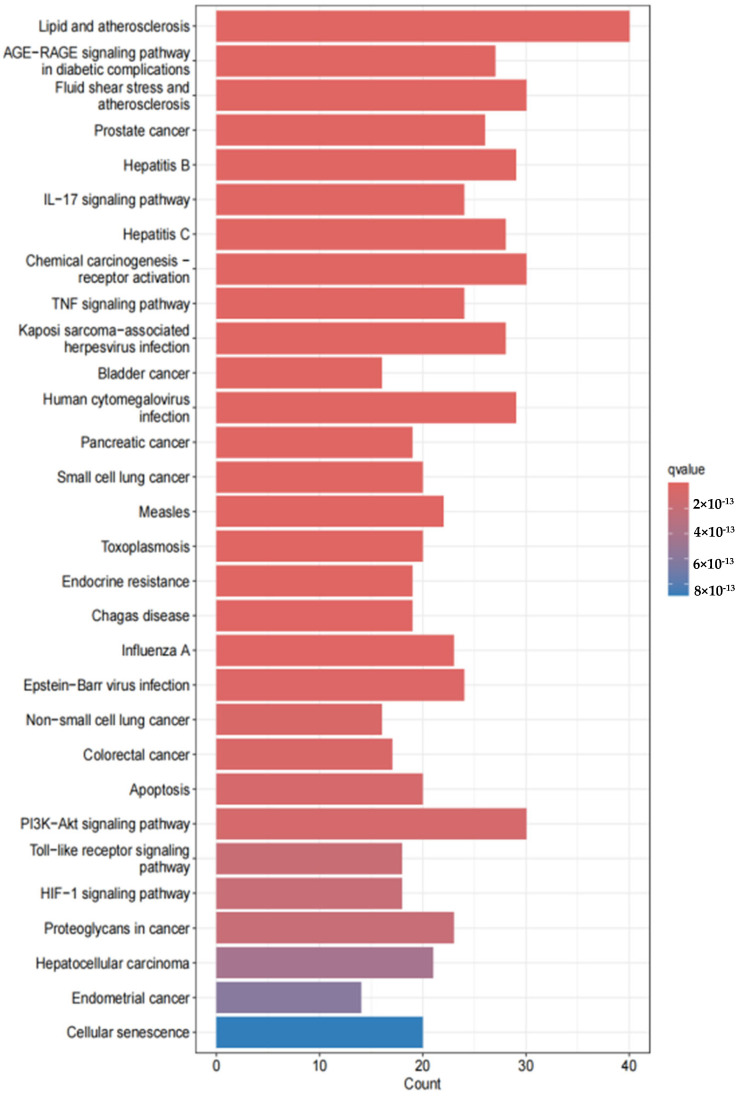
KEGG pathway enrichment analysis diagram: The x-axis represents the number of target genes enriched in the pathway, and the y-axis represents the pathway name. The top 10 significantly enriched pathways (*p* < 0.05) are shown, including the PI3K-Akt signaling pathway (related to anti-inflammatory) and cellular senescence (related to oxidative stress), indicating that EHE exerts anti-inflammatory effects through multiple pathways.

KEGG pathway screening (*p* < 0.05) identified 30 significantly enriched signaling pathways, including lipid and atherosclerosis, PI3K-Akt signaling, atherosclerosis, hepatitis B, diabetic complications, human cytomegalovirus infection, cellular senescence, small cell lung cancer, and endocrine resistance. These results are visualized in Figure 6.

## 4. Discussion

### 4.1. The Effect of EHE on the Growth Performance and Nutrient Digestibility of Calves

According to the Chinese Pharmacopoeia, EHE is a traditional Chinese herbal medicine with a clinical application history spanning hundreds of years. It exhibits stimulant and mild pharmacological activities, primarily acting on the liver and colonic systems in the treatment of human diseases. Its core bioactive components include saponins, flavonoids, soluble sugars, and polyphenols, and it possesses multiple biological activities. At present, research on EHE in calves is a blank spot, so further exploration is needed.

In the present study, calf BWG increased with increasing levels of EHE supplementation, which may be attributed to the antibacterial, anti-inflammatory, and immunomodulatory properties of its bioactive components. This is consistent with findings in broiler chickens, where dietary supplementation with 300 mg/kg total flavonoids from *E. humifusa* improved growth performance, immunity, and antioxidant capacity [19]. Additionally, phenolic compounds (e.g., *gallic acid* (GA)) enhance gastrointestinal development and growth efficiency in livestock [20] and GA supplementation improves growth performance in weaned calves [21]. The high GA content in the EHE used herein may thus contribute to the observed improvements in calf weight gain.

Our experimental results demonstrated that dry matter intake (DMI) and nutrient digestibility followed a trend consistent with body weight gain (BWG). In particular, both DMI and nutrient digestibility in Group C (the highest EHE supplementation level) were significantly higher than those in the control group (CON). Furthermore, acid detergent fiber (ADF) digestibility increased significantly with rising dietary EHE levels.

These findings align with previous studies showing that flavonoid-rich diets improve phosphorus absorption and dry matter digestibility in monogastric animals [22,23]. Correspondingly, EHE supplementation in milk replacer (MR) significantly enhanced DMI in a dose-dependent manner. Quercetin, a key flavonoid in EHE, has also been reported to promote nitrogen and crude protein (CP) digestibility [24], which may explain the upward trends—though not statistically significant—in CP, ether extract (EE), and neutral detergent fiber (NDF) digestibility observed in EHE-supplemented calves.

The observed dose-dependent increase in body weight gain and dry matter intake can be mechanistically linked to the synergistic action of EHE’s key bioactive compounds. For instance, flavonoids such as quercetin and kaempferol have been shown to enhance intestinal villus height and reduce crypt depth [25], thereby expanding the absorptive surface area in the gut. This morphological improvement likely underlies the significant increases in dry matter and acid detergent fiber digestibility we observed. Furthermore, polyphenols like gallic acid (a major component of EHE at 573.49 ng/mg) are known to strengthen the intestinal barrier function [26]. A healthier gut barrier reduces nutrient leakage and systemic inflammation, redirecting energy towards growth. The concurrent action of these compounds—improving absorptive capacity and barrier integrity—provides a compelling mechanistic explanation for the enhanced growth performance and feed efficiency in EHE-supplemented calves [27].

### 4.2. Effects of EHE on Rumen Fermentation Parameters in Calves

A stable rumen environment is critical for optimal growth in ruminants. Ruminal pH in the current study (6.01–6.17) was within the normal range (6.0–7.0) reported for preweaned calves fed milk replacer and starter feed [21], indicating that EHE supplementation did not disrupt rumen pH stability.

VFA—primarily acetic, propionic, and butyric acids—are key energy sources for ruminants, contributing −75% of metabolizable energy [28]. Acetic and propionic acids fuel ruminal microbial metabolism via distinct pathways, and A/P modulates microbial community structure, fermentation patterns, and energy utilization efficiency. For preweaned calves, propionic acid is a key energy source for hepatic gluconeogenesis. The tendency of EHE to reduce the A/P ratio (0.05 < *p* < 0.1) may indicate improved energy utilization, which is consistent with findings in preweaned calves supplemented with plant extracts [29]. In this study, EHE supplementation significantly reduced the ruminal A/P ratio in preweaned calves. This aligns with evidence that saponins alter rumen fermentation by inhibiting protozoa—specifically, protozoa phagocytose bacteria—so inhibiting protozoa reduces bacterial phagocytosis, increases bacterial counts, and ultimately lowers the A/P ratio [30]. Notably, in preweaned calves, the majority of metabolizable energy (ME) is derived from milk fat and lactose digested and absorbed in the small intestine, rather than from ruminal VFA. Although ruminal VFA production may stimulate rumen development and exert long-term positive effects on growth (e.g., promoting papillae development), their proportional contribution to the total ME supply is much lower than that in mature ruminants. This distinction is important to contextualize the observed rumen fermentation results, as EHE′s effects on VFA production are likely more relevant to rumen development than immediate energy supply in preweaned calves.

While research on calf rumen fermentation is limited, phenolic compounds like resveratrol and GA regulate rumen fermentation in other ruminants and in vitro models. For example, GA modifies ruminal microbial metabolic pathways to influence VFA production ratios, suggesting a role in balancing A/P [21]. In our study, total VFA concentrations and ruminal ammonia nitrogen levels remained within physiological ranges across all groups, indicating that EHE supplementation does not disrupt normal ruminal microbial growth or compromise the rumen environment.

### 4.3. Effects of EHE on Diarrhea and Fecal Fermentation Parameters in Calves

Neonatal calves have immature immune and digestive systems—including a rumen that is far less developed than the lower gut (e.g., small intestine). Consequently, the rumen’s contribution to total nutrient and energy supply is substantially lower than that of the small intestine during the preweaning period, making diarrhea (often linked to intestinal dysfunction) a major cause of morbidity and mortality.

The observed improvements in calf body weight gain (BWG) following *Euphorbia humifusa* extract (EHE) supplementation may partially stem from its bioactive constituents, including flavonoids and polyphenols. These compounds possess antibacterial, anti-inflammatory, hemostatic, and detoxifying properties, with documented efficacy in mitigating diarrhea in monogastric species (e.g., pigs).

Gut microbiota development and function are critical for preweaned calf health [31], as they play pivotal roles in microbial fermentation and intestinal immune regulation [32]. The flavonoids provided by EHE have a good therapeutic effect on piglet dysentery, and it is recommended to use a dose of 10 mg/mL or higher in clinical practice to achieve optimal therapeutic effects. The results of the current study are consistent with this, which may be related to the antibacterial and anti-inflammatory effects of active ingredients such as flavonoids and polyphenols in EHE.

Studies have shown that flavonoids and their metabolic derivatives can regulate intestinal barrier permeability, protect the mucosal layer, modulate the intestinal immune system, and exhibit antibacterial and anti-inflammatory properties [33]. Adding 400 mg/kg gallic acid to the diet reduced the diarrhea rate of weaned piglets [34]. There are also studies indicating that gallic acid plays a positive role in the barrier function of porcine intestinal epithelial cells (IPEC-J2) by inhibiting the NF-κB signaling pathway to alleviate intestinal inflammation [28]. Ma et al. [35] found that adding 500 mg/kg rutin to the diet increased the G/F ratio of weaned piglets by improving intestinal morphology. Meanwhile, rutin alleviates diarrhea by enhancing intestinal barrier function, which may be related to reducing intestinal inflammation and improving the antioxidant capacity and cecal microbiota composition of weaned piglets. Studies have shown [36] that caffeic acid can improve intestinal morphology and gut microbiota structure; enhance antioxidant capacity, immune function, and intestinal tight junctions; strengthen intestinal barrier function; and promote growth.

Numerous studies have shown that short chain VFA in the gut have a positive impact on gut health. In this experiment, adding EHE to preweaned calf MR reduced the fecal score and diarrhea rate, and did not affect the intestinal pH and ammonia nitrogen concentration. However, adding EHE to MR significantly increased the total volatile fatty acid concentration and acetic acid ratio of calves before weaning for 6–36 days. The flavonoids (such as quercetin and kaempferol) and gallic acid in the EHE have the ability to regulate the gut microbiota. In addition, phenolic acid components in EHE can inhibit inflammation-related bacterial genera (e.g., *Enterococcus*) in Firmicutes, optimize bacterial community structure, and further promote acetic acid production [37].

For example, adding EHE to MR can enhance the activity of fiber-degrading bacteria, increase the total VFA concentration in feces (such as increasing acetic acid concentration by 8.48–18.61%), optimize rumen fermentation parameters, and indirectly reduce diarrhea [38,39]. The EHE used in the present study contains caffeic acid as a major active constituent [40]. When acting synergistically with other EHE bioactive components (e.g., *flavonoids, gallic acid*), caffeic acid contributes to reduced diarrhea incidence, enhanced immune function, and improved growth performance in preweaned calves.

### 4.4. The Effect and Mechanism of EHE on the Anti-Inflammatory Performance of Serum in Preweaned Calves

Preweaned calves (0–60 days old) have immature intestinal and immune systems, making them prone to inflammatory responses triggered by weaning stress, pathogenic infections (e.g., *Escherichia coli*, *rotavirus*), or feed antigens. These inflammatory responses are characterized by elevated serum pro-inflammatory factors, reduced antioxidant capacity, and impaired growth or survival. Plant-derived bioactive compounds can modulate inflammatory factor secretion via nuclear factor activation and transcription factor regulation, exerting anti-inflammatory effects [41].

Cytokines are key regulators of inflammatory responses, with pro-inflammatory factors (e.g., tumor necrosis factor-α [TNF-α], interleukin-6 [IL-6], interleukin-8 [IL-8]) and anti-inflammatory factors (e.g., interleukin-10 [IL-10]) playing functionally opposing roles. In the present study, EHE supplementation alleviated diarrhea symptoms and reduced its incidence, an effect likely mediated by the downregulation of serum IL-6 and IL-8 and the upregulation of IL-10. Optimal anti-diarrheal and immunomodulatory effects were observed in Groups B (800 mg/d EHE) and C (1200 mg/d EHE), consistent with the serum cytokine profiles (Table 9). The age-specific reduction in IL-6 and IL-8 (only at 37–66 d) may be related to immune system maturity. At 6–36 d, the calf immune system is immature, and EHE’s anti-inflammatory effect may be insufficient to counteract basal inflammation; at 37–66 d, as the immune system matures, EHE can more effectively regulate pro-inflammatory cytokine secretion, leading to reduced IL-6 and IL-8.

This aligns with evidence that *E. humifusa* flavonoids inhibit inflammation by modulating these cytokines, consistent with broader findings that flavonoids (e.g., *baicalin, quercetin*) reduce pro-inflammatory cytokines (TNF-α, IL-6) and increase IL-10 [42]. Mechanistically, flavonoids block NF-κB nuclear translocation, reducing pro-inflammatory factor transcription [43]. Quercetin, for example, inhibits TNF-α production [44] and downregulates PI3K-AKT signaling to reduce expression of PI3K, TNF-α, AKT1, IL-1β, and IL-6 [45]. GA, a major polyphenol in EHE, also reduces TNF-α and IL-6 concentrations by regulating linoleic acid and ascorbic acid metabolism [46]. Similarly, trans-ferulic acid—a phenolic acid with antioxidant and immune-modulatory properties—reduces serum IL-6 by 25–30% in piglets [47], supporting the observed anti-inflammatory effects in calves. These findings indicate that EHE exerts dose-dependent anti-inflammatory activity, with mechanistic parallels to its effects in monogastrics.

### 4.5. Exploring the Anti-Inflammatory Mechanism of EHE Based on Network Pharmacology

This study demonstrated that EHE modulates serum inflammatory factors in calves. To elucidate its anti-inflammatory mechanism, network pharmacology analysis was performed. Key targets of EHE active compounds included BCL2, IL6, CASP3, EGFR, MMP9, PTGS2, TNF, and HIF1A. These targets mediate anti-inflammatory effects potentially through immune and stress responses, and bacterial proliferation/differentiation pathways.

BCL2, as an anti-apoptotic protein, protects intestinal epithelial cells from apoptosis by inhibiting mitochondrial cytochrome C release, reducing caspase-9 and caspase-3 activation. In the calf diarrhea model, downregulation of BCL2 expression is positively correlated with the degree of intestinal mucosal damage [48]. IL6, as pro-inflammatory cytokine, activates the JAK/STAT3 pathway. Increased IL6 levels correlate with diarrhea severity in *E. coli*-infected calves; gallic acid alleviates symptoms by inhibiting IL6. IL6 also enhances B-cell differentiation, antibody secretion, and immune defense, aiding pathogen clearance and reducing infection-induced inflammation and oxidative stress [49]. CASP3 is a key executing enzyme in cell apoptosis, and its activation leads to the shedding of intestinal epithelial cells and disruption of barrier function. Flavonoids, such as baicalin, inhibit CASP3 activity, reduce cell apoptosis, and promote intestinal mucosal repair [50]. EGFR activation promotes the proliferation and migration of intestinal epithelial cells, accelerating injury repair. In the calf diarrhea model, the EGFR signaling pathway is inhibited, while rutin can promote tight junction protein expression and enhance intestinal barrier by activating EGFR [51]. MMP9 is involved in extracellular matrix degradation, and overexpression leads to the destruction of intestinal mucosal structure. In the calf transport stress model, elevated MMP9 activity is associated with increased intestinal permeability, and quercetin can alleviate inflammation by inhibiting MMP9 activity [52]. PTGS2 (COX-2) catalyzes the synthesis of PGE2 and exacerbates inflammatory reactions. Rutin alleviates intestinal inflammation in calves by inhibiting PTGS2 activity and reducing PGE2 production [53]. TNF-α is a core pro-inflammatory cytokine amplifying inflammation via NF-κB activation. Gallic acid (1 g/kg feed) significantly reduces serum TNF-α levels in calves by inhibiting NF-κB nuclear translocation.

Trans-ferulic acid can significantly reduce the levels of pro-inflammatory cytokines in calf serum. For example, in the lipopolysaccharide (LPS)-induced inflammation model, trans-ferulic acid reduces the secretion of TNF-α and IL-6 by inhibiting the NF-κB signaling pathway [53].

GO and KEGG enrichment analysis of these key targets confirmed their significant involvement in inflammatory response processes. GO analysis indicated EHE modulates immune and stress responses, bacterial proliferation/differentiation, and cell-structure-related pathways. KEGG analysis revealed enrichment in pathways related to cancer, liver injury, apoptosis, viral infection, inflammation, immune regulation, cardiovascular/metabolic disease, cell proliferation/death regulation, carcinogenesis/drug resistance, and hypoxia/microenvironment regulation. This suggests EHE’s anti-inflammatory effect involves interplay with factors like cancer and liver injury, while mitigating oxidative stress represents a common therapeutic strategy.

Combining network pharmacology and molecular docking, this study systematically reveals that EHE alleviates calf diarrhea through a multi-component, multi-target, and multi-pathway network. Its core mechanism involves immunosuppressive, anti-inflammatory, antiviral, and antitumor activities, providing a theoretical basis for EHE’s clinical application.

## 5. Conclusions

(1) Apparent performance: 1200 mg/head/day *Euphorbia humifusa* extract (EHE) significantly increased calf final body weight and DMI, DM, and ADF digestibility. CP, EE, and NDF digestibility showed positive trends. And EHE significantly reduced the symptoms of diarrhea.
(2) Serum biomarkers: EHE (800/1200 mg) reduced serum IL-6/IL-8 at 60 d and dose-dependently elevated IL-10 (1200 mg), confirming age-dependent immunomodulation.
(3) Network Pharmacology: EHE targeted BCL2, IL6, CASP3, EGFR, MMP9, PTGS2, TNF, HIF1A via multi-target mechanisms. Enriched pathways included immune stress/apoptosis. NF-κB inhibition mediated anti-inflammatory/antiviral effects and barrier protection.

## Figures and Tables

**Table 1:** List of active ingredients in EHE.

Items	Ingredients	Contents
Saponin	Glycoside compounds	11%
Total Flavonoids	Flavonoids	3.23 mg/g
Saccharides	Maltose	22.49 μg/mg
	Glucose	12.66 μg/mg
	Fructose	5.69 μg/mg
	L-Fucose	2.53 μg/mg
Polyphenol	Gallic acid	573.49 ng/mg
	Xanthophyll	45.64 ng/mg
	Quercetin 3-β-D-glucoside	26.86 ng/mg
	Rutin	21.02 ng/mg
	Trans-Ferulic acid	11.00 ng/mg
	Caffeic acid	9.50 ng/mg
	P-Hydroxycinnamic acid	7.83 ng/mg
	3,4-Dihydroxybenzoic acid	7.35 ng/mg
	Catechin	5.87 ng/mg
	Apigenin	5.83 ng/mg
	Kaempferol-3-O-glucoside	3.57 ng/mg
	Luteolin	3.37 ng/mg
	Quercetin	2.66 ng/mg
	Vanillin	2.41 ng/mg

**Table 2:** Experimental design.

Groups	Diets	
	**3–30 Days of Age**	**30–60 Days of Age**
CON	Milk Replacer (0 mg)	Milk Replacer (0 mg)
Group A	Milk Replacer (400 mg)	Milk Replacer (800 mg)
Group B	Milk Replacer (800 mg)	Milk Replacer (1200 mg)
Group C	Milk Replacer (1200 mg)	Milk Replacer (2400 mg)

Calves were fed milk replacer twice daily according to the following schedule: from days 3 to 7, 4 L/day; days 7 to 14, 6 L/day; days 15 to 25, 7 L/day; days 25 to 35, 8 L/day; days 36 to 43, 7 L/day; days 44 to 51, 6 L/day; and days 52 to 57, 4 L/day. Weaning occurred from days 58 to 63, during which the daily volume was reduced by 1 L each day until complete cessation.

**Table 3:** Nutrient levels of MR and starter (air-dry basis).

Items	MR	Starter
Ingredient, %		
Corn maize	-	40
Soybean meal	-	25
Wheat bran	-	15
Expanded soybean	-	10
Wheat middlings		8.6
CaHPO_4_	-	0.5
Nacl	-	0.9
Nutrient		
CP, %	23.58	22.58
EE, %	15.99	5.03
Ash, %	5.02	3.73
NDF, %	3.98	16.09
ADF, %	2.01	7.07
Ca, %	0.85	0.82
P, %	0.54	0.56
GE, MJ/kg	17.15	15.35

The premix provided the following per kg of the starter: ① VA 11,000 IU, VD 3000 IU, VE 50 IU, Fe 84 mg, Cu 11 mg, Mn 40 mg, Zn 100 mg, Se 0.3 mg, I 0.8 mg, Co 0.4 mg. ② Nutrient levels were measured values.

**Table 4:** Fecal score system.

Levels	Appearance	Score
Normal	Firm but not hard, original form is distorted slightly after dropping to floor and settling	0
Soft	Does not hold form, piles but spreads slightly	1
Runny	Spreads readily	2
Watery	Liquid consistency, splatters	3

**Table 5:** Effects of EHE on growth performance of preweaned calves.

Items	Groups				SEM	*p*-Value			
	CON	Group A	Group B	Group C		T	D	T × D	L
Initial weight, kg	41.5	40.8	43.3	43.1	0.61	0.384			
Final weight, kg	86.5 ^b^	89.7 ^ab^	91.6 ^ab^	95.6 ^a^	1.42	0.015			
Total weight gain, kg	45.00 ^b^	48.88 ^ab^	48.39 ^ab^	52.50 ^a^	1.15	0.034			
ADG, g/d	738.3	794.4	793.6	842.3	17.15	0.211	<0.010	0.056	0.147
DMI, kg	1117.16 ^b^	1192.85 ^ab^	1159.17 ^b^	1321.40 ^a^	26.84	0.042	<0.010	0.046	0.156
F/G	1.54	1.51	1.47	1.58	0.03	0.734	0.091	0.218	0.145
Nutrient digestion rate, %									
DM	69.15 ^b^	69.92 ^b^	72.09 ^ab^	74.31 ^a^	0.73	0.043			
CP	72.52	69.13	74.29	75.18	1.29	0.381			
EE	80.04	81.20	83.05	82.33	1.27	0.868			
NDF	41.07	42.51	46.56	47.72	1.17	0.132			
ADF	28.98 ^b^	32.58 ^ab^	34.09 ^ab^	35.85 ^a^	0.95	0.056			

ADG = average daily gain, F/G = Feed conversion rate, DM = Dry matter, CP = Crude protein, EE = Ether extract, NDF = Neutral detergent fiber, ADF = Acidic detergent fiber. CON: MR without EHE; Group A: MR supplemented with 400 mg/d EHE (3–30 d) and 800 mg/d EHE (31–60 d); Group B: MR supplemented with 800 mg/d EHE (3–30 d) and 1200 mg/d EHE (31–60 d); Group C: MR supplemented with 1200 mg/d EHE (3–30 d) and 2400 mg/d EHE (31–60 d). Different letters in the same column indicate significant differences (*p* < 0.05), while no letters indicate insignificant differences (*p* > 0.05). Among the variables, T is the treatment group, D is age; T × D corresponds to the *p*-value associated with the interaction between treatment and age, while L is the contrast test conducted to assess the linear trend of treatment.

**Table 6:** Effects of EHE on rumen fermentation parameters in preweaned calves.

Items	Groups				SEM	*p*-Value
	CON	Group A	Group B	Group C		
pH	6.08	6.01	6.06	6.17	0.09	0.956
TVFAs, mmol/L	103.78	99.16	109.90	105.67	3.62	0.351
Acetate, %	53.25	47.91	54.29	50.68	1.77	0.264
Propionic acid, %	33.50	35.05	40.87	37.25	1.63	0.162
A/P	1.63	1.54	1.29	1.22	0.80	0.079
Isobutyric acid, %	0.45	0.46	0.51	0.50	0.26	0.082
Butyric acid, %	12.04	11.39	9.08	11.35	1.08	0.393
Isovaleric acid, %	0.68	0.58	0.68	0.67	0.13	0.799
Valeric acid, %	3.86	3.78	4.47	5.21	0.33	0.168
NH_3_-N, mg/dL	13.37	15.97	14.48	16.64	0.98	0.680

Each feed material was tested with 3 replicates, and each replicate included 3 parallel samples. In the same column, different letters indicate significant differences (*p* < 0.05), whereas the absence of letters indicates no significant differences (*p* > 0.05).

**Table 7:** Effects of EHE on fecal score and diarrhea rate in preweaned calves.

Items	Groups				SEM	*p*-Value			
	CON	Group A	Group B	Group C		T	D	T × D	L
Fecal score									
6–36	2.73 ^a^	2.22 ^ab^	1.75 ^bc^	1.52 ^c^	0.21	0.003			
37–66	2.32	2.06	1.78	1.37	0.25	0.059			
Total period	2.55 ^a^	1.48 ^ab^	1.76 ^bc^	1.48 ^c^	0.16	0.003	<0.010	<0.010	0.851
Diarrhea frequency, %									
6–36	31.80	29.60	27.41	17.44	1.62	0.060			
37–66	27.01	27.83	22.60	15.40	1.65	0.063			
Total period	29.41 ^a^	28.72 ^a^	25.01 ^b^	16.42 ^c^	3.01	0.008	<0.010	0.060	0.768

There were 3 replicates for each feed material and 3 parallel replicates for each replicate. Different letters in the same column indicate significant differences (*p* < 0.05), while no letters indicate insignificant differences (*p* > 0.05). T is the treatment group, D is the age, T × D = *p*-value of interaction between treatment and age, L = contrast test for linear trend of treatment.

**Table 8:** Effects of EHE on fermentation parameters of feces in preweaned calves.

Items	Groups				SEM	*p*-Value			
	CON	Group A	Group B	Group C		T	D	T × D	L
pH									
6–36	7.14	7.09	7.20	7.18	0.14	0.698			
37–66	7.28	7.20	7.26	7.37	0.09	0.961			
Total period	7.21	7.09	7.23	7.28	0.21	0.538	0.398	0.633	0.478
Total VFA, μmol/g									
6–36	27.00	36.49	35.18	40.73	2.03	0.233			
37–66	37.19	27.41	34.54	34.16	2.60	0.639			
Total period	32.10	31.95	34.86	37.45	1.67	0.869	0.495	0.398	0.578
Acetic acid, %									
6–36	17.14	25.09	25.12	26.95	1.27	0.056			
37–66	23.00	16.81	22.27	20.97	1.81	0.662			
Total period	20.07	20.95	23.70	23.96	1.03	0.396	0.637	0.379	0.472
Propionic acid, %									
6–36	6.05	8.81	8.47	9.58	0.63	0.406			
37–66	7.35	6.64	7.02	9.93	0.58	0.247			
Total period	6.71	7.73	7.75	9.76	0.86	0.827	0.485	0.583	0.893
Isobutyric acid, %									
6–36	0.53 ^b^	0.57 ^b^	0.53 ^b^	1.23 ^a^	0.09	0.002			
37–66	1.39 ^a^	0.81 ^c^	0.94 ^bc^	1.23 ^ab^	0.08	0.006			
Total period	0.96 ^a^	0.69 ^b^	0.74 ^b^	1.23 ^a^	0.13	0.011	<0.010	<0.010	0.746
Butyric acid, %									
6–36	2.76	3.58	3.21	4.50	0.28	0.235			
37–66	3.93	2.91	3.26	3.87	0.25	0.483			
Total period	3.34	3.24	3.24	4.19	0.51	0.255	0.857	0.495	0.875
Isovaleric acid, %									
6–36	0.45	0.51	0.50	0.67	0.08	0.159			
37–66	0.86 ^a^	0.65 ^b^	0.87 ^a^	0.75 ^ab^	0.08	0.016			
Total period	0.66 ^ab^	0.58 ^ab^	0.69 ^a^	0.71 ^a^	0.04	0.027	<0.010	<0.010	0.758
Valeric acid, %									
6–36	0.37 ^b^	0.38 ^b^	0.47 ^b^	0.67 ^a^	0.05	0.002			
37–66	0.31 ^c^	0.45 ^b^	0.53 ^b^	0.68 ^a^	0.07	0.002			
Total period	0.34 ^b^	0.42 ^ab^	0.53 ^a^	0.56 ^a^	0.05	0.047	<0.010	<0.010	0.656
Ammonia nitrogen, mg/g feces									
6–36	3.48	3.64	3.53	3.70	0.62	0.874			
37–66	3.56	3.60	3.58	3.79	0.28	0.235			
Total period	3.52	3.62	3.56	3.75	0.46	0.961	0.754	0.584	0.685

Each feed material had 3 replicates, with 3 parallel subsamples per replicate. Total VFA is expressed as concentration (μmol/g feces); acetic acid, propionic acid, isobutyric acid, butyric acid, isovaleric acid, and valeric acid are expressed as molar percentage (%). In the same column, data denoted by different letters indicate significant differences (*p* < 0.05), while those without letter annotations are not significantly different (*p* > 0.05). T is the treatment group, D is the age, T × D = *p*-value of interaction between treatment and age, L = contrast test for linear trend of treatment.

**Table 9:** Effect of EHE on serum cytoinflammatory factors in preweaned calves.

Items	Treatment				SEM	*p*-Value			
	CON	Group A	Group B	Group C		T	D	T × D	L
TNF-α, pg/mL									
6–36	146.68	148.01	124.84	135.89	4.13	0.095			
37–66	139.28	123.54	135.16	133.29	4.56	0.684			
Total period	143.51	135.78	128.97	134.40	3.06	0.447	0.271	0.925	0.378
IL-6, pg/mL									
6–36	309.97	317.94	298.85	304.79	6.35	0.768			
37–66	312.48 ^a^	273.48 ^b^	266.11 ^b^	292.00 ^ab^	6.31	0.028			
Total period	311.22	293.24	285.76	298.39	4.79	0.297	0.060	0.766	0.638
IL-8, pg/mL									
6–36	62.79	61.34	60.53	58.15	1.89	0.903			
36–66	66.34	56.88	55.15	57.96	1.66	0.075			
Total period	64.57	58.55	58.38	58.04	1.24	0.204	0.238	0.605	0.946
IL-10, pg/mL									
6–36	127.64	130.61	123.38	136.06	3.05	0.473			
37–66	119.09	124.11	116.40	132.61	3.97	0.558			
Total period	122.75	127.00	120.59	134.53	2.51	0.198	0.465	0.844	0.867

In each column, data labeled with distinct letters signify significant differences at the *p* < 0.05 level, whereas unmarked data indicate non-significance (*p* > 0.05). T = treatment group, D = age, T × D = *p*-value of interaction between treatment and age, L = contrast test for linear trend of treatment.

## Data Availability

Data presented in the study are available upon request to the corresponding author.

## Abbreviations

The following abbreviations are used in this manuscript:

EHE	*Euphorbia humifusa* extract
ADG	Average dairy gain
A/P	Acetate-to-propionate ratio
BWG	Body Weight Gain
DAI	Disease activity index
DMI	Dry matter intake
F/G	Feed conversion rate
NDF	Neutral detergent fiber
ADF	Acidic detergent fiber
TNF-α	Tumor necrosis factor-α
IL-6	Interleukin-6
IL-8	Interleukin-8
IL-10	Interleukin-10
DM	Dry matter
CP	Crude protein
EE	Ether extract
